# Differential Diagnosis of Landau-Kleffner Syndrome Versus Post Encephalitis Syndrome in a 13-year-old Boy With Autism Spectrum Disorder

**DOI:** 10.7759/cureus.9385

**Published:** 2020-07-25

**Authors:** Bhavani G Murugesan, Abtin Jafroodifar, Arayamparambil C Anilkumar, Luba Leontieva

**Affiliations:** 1 Psychiatry and Behavioral Sciences, State University of New York Upstate Medical University, Syracuse, USA; 2 Radiology, State University of New York Upstate Medical University, Syracuse, USA; 3 Neurology, State University of New York Upstate Medical University, Syracuse, USA; 4 Psychiatry, State University of New York Upstate Medical University, Syracuse, USA

**Keywords:** landau-kleffner syndrome, childhood epilepsy, autism, tick-born encephalitis

## Abstract

Landau-Kleffner syndrome (LKS) is a rare childhood neurological condition that causes developmental regression, loss of language skills and abnormal electroencephalogram (EEG) patterns. Its etiology is unknown. This report describes a case of a 13-year-old boy who at 3.5 years of age was bitten by a tick. Two months thereafter, he began losing previously acquired developmental and language skills, and developed seizures. The seizures subsided with valproic acid treatment, but the developmental delays persisted. Family history and disease progression reports obtained from the patient’s father revealed that the patient displayed repetitive behaviors prior to the age of three. Clinical observation also showed the patient having numerous repetitive vocalizations and movements along with difficulty with switching sets. His developmental age at the time of presentation was determined to be 3 to 4 years of age. During the course of diagnostic testing, we were able to rule out tick-borne encephalitis (TBE) and rule in LKS in a premorbidly autistic child. This case describes the similarities between the three conditions and the diagnostic investigations used to arrive at a final diagnosis.

## Introduction

Landau-Kleffner syndrome (LKS)

In 1957, a study published in Neurology described the coupling of aphasia and seizures in six children. All of them developed aphasia. Some of them suffered from “absence” seizures, including partial, myoclonic, and absence seizures [1]. Originally called “acquired aphasia with a convulsive disorder,” this set of symptoms was later named (after the authors of the 1957 report) as LKS. Still considered a rare disorder, LKS affects children roughly between the ages of 2-8 years of age. Several studies have found that an arginine to histidine mutation at site 518 in the GRIN2A gene is highly correlated with LKS and other epilepsy-aphasia syndromes [2]. Boys are more likely to be affected and the syndrome is associated with partial penetrance and autosomal dominant pattern of inheritance. However, the etiology of LKS in all patients remains unknown.

LKS is most closely associated with acquired aphasia. At the time of onset, a child will present with symptoms of auditory verbal agnosia. Seizures are noted in 75%-80% of the cases with cognitive impairment, memory disorders, and global regression in behavior, as well as hyperactivity. Patients who develop aphasia before 4 years of age with accompanying speech delay and lack of fluctuations in electrical status epilepticus during sleep (ESES) have a poor prognosis. In a review of LKS patients who had a ten-year history, only 18% completely recovered their language skills and 64% had mental retardation. Electroencephalogram (EEG) findings in patients showed regional spikes in the fronto-, centro-, or posterior-temporal areas of the brain in all patients. Other characteristic EEG findings include continuous and diffuse slow spikes and waves, mostly at 1.5-2.5 Hz, immediately after the patient falls asleep. These patterns continue through all the slow wave-sleep stages [3].

Treatment for LKS has included anti-epileptic drugs (AED) with corticosteroids. Studies show that the use of AEDs alone does not improve the aphasia. Valproate has been used to prevent seizures. Sulthiame and clobazam have helped with the aphasia [1].

Tick-borne encephalitis (TBE)

In vast regions of Europe and Russia and the Far East, the tick-borne encephalitis virus (TBEV) of the genus flavivirus and the family Flaviviridae is responsible for a major tick-borne transmitted disease. TBEV has three subtypes - European, Siberian, and Far-Eastern of which the Far-Eastern subtype is the most lethal [4]. Ixodes ricinus is the known ixodid vector for the European subtype and I. Persulcatus carries the Siberian and Far-Eastern subtypes [5]. TBEV perpetuates its life cycle between the ixodid tick and wild animals, especially rodents and ticks can transmit the virus to many animal species. Thus, TBE can not only occur from tick bites but also from the consumption of unpasteurized milk.

TBE develops in a small percentage (2%-25%) of those infected with TBEV. However, the sequalae of symptoms can be severe and include neurological issues. TBE is biphasic in the European subtype and monophasic in the other two subtypes [4]. Influenza-like symptoms appear in the first phase. The incubation period for all three subtypes is 7-14 days. In the first phase, patients complain of fever, headache, malaise, and myalgia. In the second phase, patients present with central nervous system (CNS) issues. About 40%-50% of patients present with aseptic meningitis or meningoencephalitis and another 10% to 15% present with meningoencephalomyelitis. Of those who recover, a considerable number of patients continue to experience cognitive dysfunction, gait ataxia, memory deficits, temporary hearing impairment, and balance disorders [6]. These chronic conditions are most notable in TBE caused by the Siberian subtype [4].

Diagnosis of TBE in the first phase can be done with cell culture for the virus from the blood or cerebro-spinal fluid (CSF). In the second phase, TBEV-specific IgG and IgM antibodies can be detected [7]. In confirmed TBE patients, a review of magnetic resonance imaging (MRI) findings indicates involvement of the thalami, basal ganglia, cerebellum, and the anterior horns of the spinal cord [8]. Humoral responses include the release of chemokines, cytokines, T cell (T helper cell type 1 (Th1), Th17), and B cell responses. Chemokines, cytokines (tumor necrosis factor-alpha (TNF-alpha), interleukin 1 alpha (IL-1alpha), and interleukin-6 (IL-6)) and Th1 are found in the CSF during the second phase of the illness. Th17 and B cell responses are concentrated in the serum. Studies show that CNS involvement occurs in those who failed to mount an effective antibody response to prevent the virus from traveling across the blood-brain barrier. Cell-mediated immune responses play a vital role in the development of neurological issues and a high viral load is required for neurological pathogenesis.

Effective vaccines are available for TBE but there are no curative treatments. Three doses of the vaccine are required within the first year of birth followed by repeat vaccinations every three to five years to maintain immunity [9].

Autism-spectrum disorder (ASD)

ASD is a well-known neurodevelopmental disorder characterized by limited social attention, impaired social communication, and restricted and repetitive behaviors [10]. Children are usually diagnosed with ASD in the first two years of life. They achieve early language skills but begin to regress in language and communication skills somewhere before the age of two. ASD affects boys more often than girls. Affected children fail to exhibit empathy, resist change, and require a high degree of consistency. Apraxia and dyspraxia are often associated with ASD. Children do not respond when their names are called, speak in monosyllabic words and phrases, regress in their language skills, and communicate with gestures instead of words. While the causative reason for ASD remains unknown, it is generally believed that genetic factors play a heavy role. It is hypothesized that an abnormal gene is expressed in early fetal development [11]. This theory is supported by the high risk for ASD between siblings and the higher prevalence among males. The diagnosis of ASD can be problematic as other intellectual disability disorders manifest similar clinical findings.

## Case presentation

A 13-year-old male with behavioral difficulties was brought to the outpatient psychiatry clinic of this hospital by his father. The patient was prone to hitting, repetitive behaviors, and nocturnal bed wetting. Patient refused to follow directions at home and in the clinic. He had a habit of biting himself. The father sought diagnostic clarification and treatment. Family history revealed no incidences of hereditary, seizure, intellectual disability, or psychiatric disorders. The mother was 25 years old when she gave birth after suffering several miscarriages and an abortion. Patient was born full-term, delivered vaginally, and weighed 3 kgs (6.6 lbs.) at birth. He was breast fed for five months.

The patient started to hold his head at 1.5 months and began walking at 12 months of age. He was able to utter 10 words as a 1-year-old. At 2.5 years, the patient gained toilet training skills. At the same time, his parents noticed repetitive behaviors and word usage. They noted that he preferred playing with wires, attaching them to each other rather than playing with toys.

As a 3.5-year-old, the patient was bitten by a tick while in a remote area in Altaiski kray, Russia. He had three days of high fever which subsided without medical treatment. Two to three months after the bite, the patient developed “petit mal seizures”, a robotic and awkward walk, gradually stopped talking, and lost already acquired toilet training skills. He was treated with valproic acid from age 4.5 to 8 years after which the seizures ceased. He regained toileting habits and his speech somewhat improved. However, his intellectual development had halted. He was under neurological and psychiatric care in Russia where he was diagnosed with LKS and treated with cortexin, nootropics, hopantenic acid, magnesium, B6, Sonopax (thioridazine), and glycine. The patient remained intellectually delayed with almost no coherent speech. He displayed repetitive behaviors, occasional aggression towards his father, and periodic enuresis. He attended special education classes and, at some point, resided in a group home in Russia.

The patient and his father relocated to the US when the patient was 12 years old. At the time of the initial encounter, patient was in a Grade 6 Special Education program. He was described as loving school, very social and enjoying cross country skiing, swimming, and other sports activities. He could dress himself, use the bathroom, clean his room, cook simple meals, eat with some utensils, shovel snow and use the computer for simple searches. He watched cartoons, but without comprehension of the content. He could not read or write and could only say one word in English and Russian. He used his father’s hand to point to what he wanted. He often engaged in his favorite activities for hours. Intellectually, he was very delayed. Psychological testing was attempted but difficult to complete due to the serious intellectual delay and poor cooperation, and his difficulty in following directions. His attention span was very short, and he required constant re-directions.

Patient was impulsive and aggressive at times towards his father, schoolteachers, and aides. His teacher’s observation questionnaire revealed a very low level of adaptive functioning in all areas. For this behavioral problem, he was prescribed risperidone, aripiprazole, clonidine, methylphenidate, and olanzapine. Clonidine made him tired and worsened the behavioral problems, and methylphenidate did not have any effect on his concentration. From a behavioral control standpoint, risperidone and subsequently olanzapine had favorable effect on abating his aggression.

Before beginning any diagnostic investigations, we corresponded with the Center for Disease Control (CDC) and confirmed that if the patient had encephalitis, he would produce immunoglobulin G (IgG) antibodies which would remain in the blood stream for 10 years. Diagnostic testing for West Siberia, Russian Spring Summer, and Far East encephalitis viruses were performed and results for all IgGs came back negative, indicating that the patient never had TBE. This ruled out TBE and its sequela as a causative agent for his developmental delay and poor mental health.

**Figure 1:**
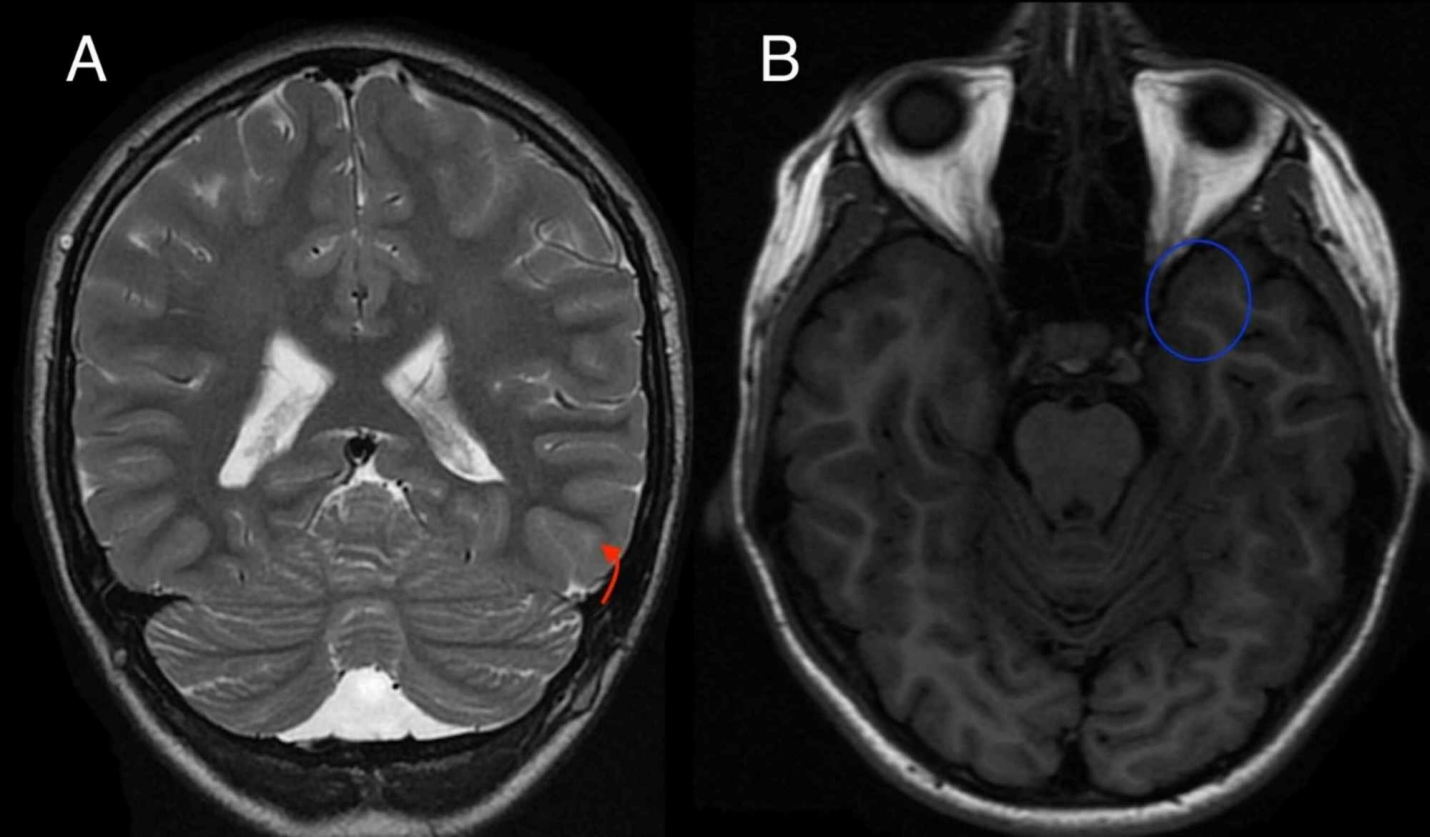
Coronal T2-weighted MR image of the brain (Figure 1A) demonstrates a focus of hyperintensity within the left temporal lobe (curved red arrow). Axial T1-weighted MR image of the brain (Figure 1B) demonstrates mild asymmetry of the hippocampal regions, with slight hyperintensity seen in the left hippocampus (blue circle).

**Figure 2:**
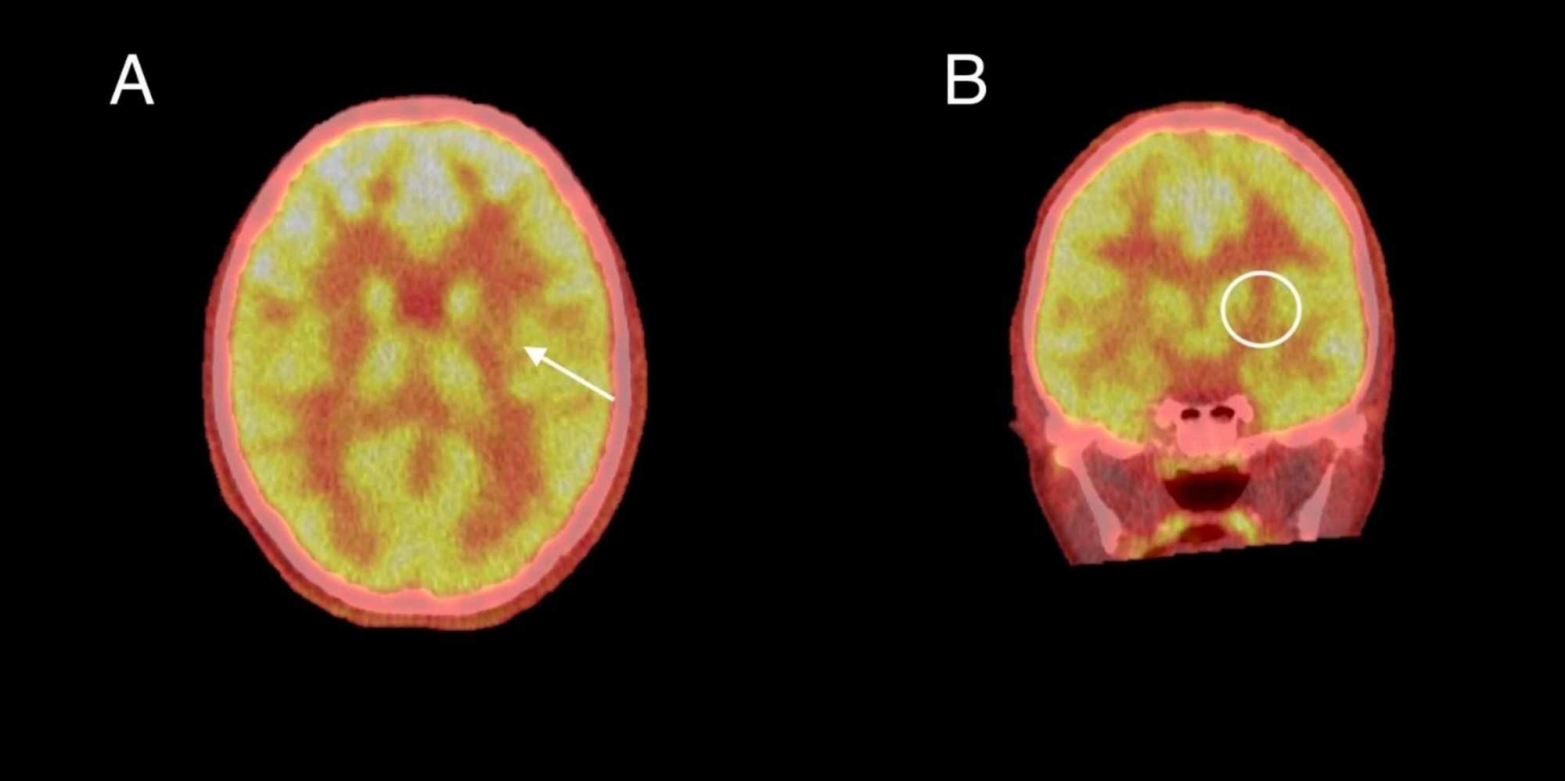
Axial coronal PET/CT fused image of the brain (Figure 2A) demonstrate asymmetric diminished activity within the left basal ganglia, as compared to the right (white arrow). This relative decreased activity is further demonstrated on the coronal PET/CT fused image of the brain (white circle in Figure 2B). PET/CT: positron emission tomography/computed tomography

An MRI without contrast, a positron emission tomography-computed tomography (PET-CT) of the brain, and a video EEG were ordered to help localize the seizure focus. A multiplanar, multisequence MRI study of the brain was performed with seizure protocol (Figure 1). A diffusion tensor imaging (DTI) study was also obtained. For the PET scan, 30 minutes following the intravenous injection of 4.4 mCi of F-18 labeled fluorodeoxyglucose (FDG), PET-CT imaging of the brain was obtained (Figure 2). This study was compared to the MRI of the brain (Figure 1). The patient's blood glucose level at time of injection was 90 mg for deciliter. The study was performed under general anesthesia. MRI was also reviewed, which showed lesions suspicious for cortical dysplasia, with thick gyri on left post frontal, as well as the temporal region.

A digital long-term video EEG monitoring study was performed both in the awake and asleep states over the course of two days and one night.

**Figure 3:**
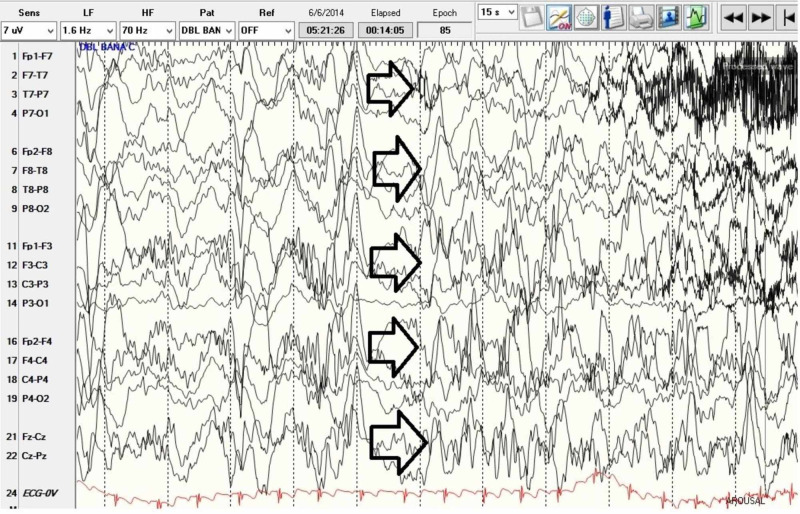
Electroencephalogram showing sleep architecture with embedded continuous generalized slow spike wave activity.

**Figure 4:**
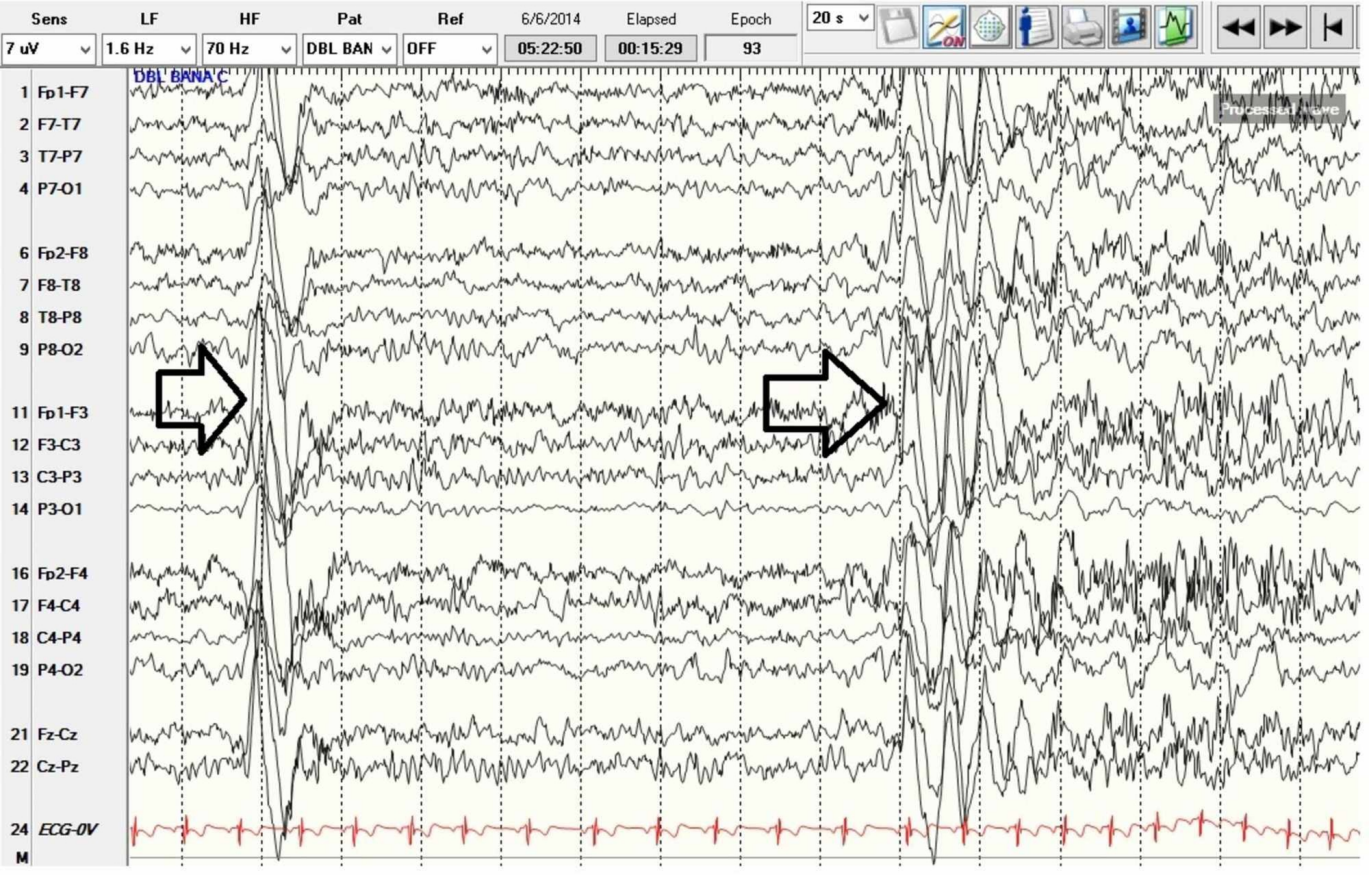
Electroencephalogram showing bursts of generalized discharges.

**Figure 5:**
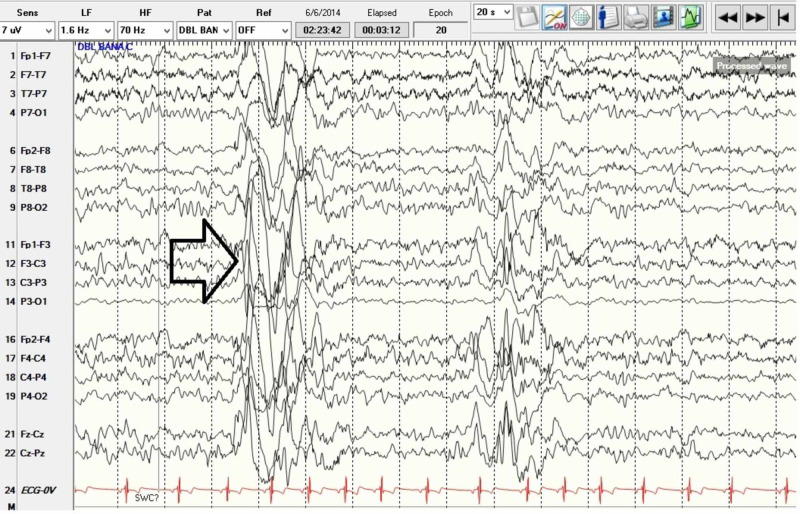
Electroencephalogram showing wakeful state with bursts of generalized epileptiform activity as well as multifocal discharges.

Patient’s EEG was abnormal due to left temporal slowing as well as left anterior, mid, posterior temporal as well as left central, parietal, occipital atypical spikes, spike and slow waves and with rare generalized bursts of spike and slow waves. This may be consistent with a diagnosis of epilepsy with potential generalized and/or multifocal onset with potential onset from left temporal, central, parietal, occipital head regions. There is augmentation of continuous runs of generalized discharges of 1-1.5 Hz suggestive of potential LKS which might account for his speech difficulties (Figures 3-5). Per neurology recommendation, the patient was started on lamotrigine for the prevention of seizures.

The patient was also referred to a psychologist to have his intelligence quotient (IQ) tested. The testing was conducted on three days: May 23, 2014, May 28, 2014, and June 6, 2014. However, the IQ testing could not be completed due to patient’s non-cooperation. Intellectual delay, very low level of adaptive functioning, and ASD was documented by the psychologist based on behavior observations, Vineland Adaptive Behavior Scales - Second Edition (VABS-II) [12], and Autism Diagnostic Interview-Revised (ADI-R) [13].

In follow up, patient was titrated up on lamotrigine to a total of 200 mg daily. For behavioral problems, he was treated with risperidone, and later olanzapine. Over time, the dosage of lamotrigine was titrated down and discontinued as the patient became seizure-free.

At the time of this report, this now 19-year-old patient remains largely non-verbal. He can verbalize two words sentences in English. He takes olanzapine 5-10 mg daily for behavioral control. He has no clinical seizures. He takes care of his basic needs and attends special education programs in school. He is interested in many sports and can perform simple computer searches.

## Discussion

TBE, LKS, and autism share several common findings. All three cause neurological changes and delays. LKS and autism affect young children. Both disorders are also more prevalent in males and symptoms appear around the age of 2-3 years. Although genetics appears to play a key role in the development of autism and LKS, the exact mechanism and causation remains unknown. In our case, the patient showed some symptoms of autism before suffering a tick bite. The lack of IgG indicated that he did not acquire TBE from the bite. However, we wonder if the bite precipitated the onset of LKS. A review of the current literature indicates that the onset of LKS symptoms has no common thread. One report discussed a child who developed LKS after a fever with convulsions. Another was diagnosed after experiencing a stressful event related to his parents [14].

Whether this patient’s regression in language was a progression of autism or brought about by LKS or a combination of both is a question to be considered. Diagnosis was difficult given the overlap of clinical signs and symptoms in all three conditions. It is also possible for all three disorders to coexist in an unfortunate individual. In our case, we were able to rule out encephalitis as a possible cause of developmental delay and neurological symptoms such as seizures. This left us with two conditions to consider: LKS and ASD. The thorough clinical diagnostic investigation revealed that our patient has symptoms of ASD prior to developing regression in language and skills. LKS was ruled in as the patient had EEG showing epileptiform activity in sleep, positive findings on MRI of the brain, and PET brain study all indicated left lateralization (left temporal, left hippocampal, and left basal ganglia areas). The findings suggest that the language delay could be the result of localization of pathology in the left side of the brain, the language area. Table 1 summarizes a comparison between LKS and TBE.

**Table 1:** Comparison of Landau-Kleffner syndrome to tick-borne encephalitis. CSF: cerebro-spinal fluid; TBEV: tick-borne encephalitis virus; IgG: immunoglobulin G; IgM: immunoglobulin M; Th1: T helper cell type 1; Th17: T helper cell type 17; TNF-alpha: tumor necrosis factor-alpha; IL-1alpha: interleukin 1 alpha; IL-6: interleukin-6

	Landau-Kleffner Syndrome	Tick-Borne Encephalitis
Mode of transmission	Idiopathic; possible genetic predisposition (partial penetrance and autosomal dominant pattern of inheritance)	Tick bite, unpasteurized milk
Prevalence	Children; with male predominance between the ages of 2-8 years.	No preference
Testing	EEG for focal and or generalized epileptiform activity and continuous spikes and waves in slow wave sleep.	First phase - Cell culture for virus in blood or CSF. Second phase - Antibodies in serum: TBEV-specific IgG and IgM antibodies; Th17 and B cell response. Antibodies in CSF: TNF-alpha, IL-1alpha, IL-6 and Th1. Chronic - Serum IgG antibodies to virus can be present for 10 years
Clinical findings	Aphasia, seizures, impairment in cognition, memory, and global regression in behavior.	First phase - Influenza-like symptoms of fever, headache, malaise and myalgia. Second phase - features of aseptic meningitis, meningoencephalitis or meningoencephalomyelitis. Chronic - Cognitive dysfunction, gait ataxia, memory deficits, transient hearing impairment, balance problems.
Prevention	None	Vaccine
Treatment	Anti-epileptic drugs (valproate, sulthiame and clobazam), corticosteroids.	Supportive care and symptom control.

## Conclusions

This case presented a diagnostic challenge in a patient with severe intellectual delay and neurological symptoms. Diagnostic investigations ruled out TBE as a potential cause of developmental delay and seizures. Based on diagnostic imaging test results, LKS was ruled in. Based on the patient’s history of repetitive behaviors, non-animated objects play, difficulties shifting sets, intellectual delay, and specific interactions with the father, it was determined that the patient met the criteria for ASD. It was concluded that the patient had ASD with LKS.
